# Data Analytics of a Wearable Device for Heat Stroke Detection

**DOI:** 10.3390/s18124347

**Published:** 2018-12-09

**Authors:** Shih-Sung Lin, Chien-Wu Lan, Hao-Yen Hsu, Sheng-Tao Chen

**Affiliations:** Department of Electrical and Electronic Engineering, Chung Cheng Institute of Technology, National Defense University No. 75, Shiyuan Rd., Daxi District, Taoyuan City 33551, Taiwan; chienwulan@gmail.com (C.-W.L.); shihaoyen@gmail.com (H.-Y.H.); iiccanffly@gmail.com (S.-T.C.)

**Keywords:** heat stroke, filtering algorithm, physiological parameters, exercise experiment

## Abstract

When exercising in a high-temperature environment, heat stroke can cause great harm to the human body. However, runners may ignore important physiological warnings and are not usually aware that a heat stroke is occurring. To solve this problem, this study evaluates a runner’s risk of heat stroke injury by using a wearable heat stroke detection device (WHDD), which we developed previously. Furthermore, some filtering algorithms are designed to correct the physiological parameters acquired by the WHDD. To verify the effectiveness of the WHDD and investigate the features of these physiological parameters, several people were chosen to wear the WHDD while conducting the exercise experiment. The experimental results show that the WHDD can identify high-risk trends for heat stroke successfully from runner feedback of the uncomfortable statute and can effectively predict the occurrence of a heat stroke, thus ensuring safety.

## 1. Introduction

According to the 2017 global climate report published by the National Oceanic and Atmospheric Administration of the United States [1], the global temperature in 2017 reached the third highest recorded in history. Moreover, the global temperature was also found to increase 0.07 °C every ten years. These findings indicate an evident trend of global warming occurring in recent decades. This trend has had a significant impact on Taiwan as well. The main island of Taiwan is located on the Tropic of Cancer. The northern part of Taiwan falls within the subtropical zone whereas the southern part is within the tropical climate zone. Nevertheless, both parts of Taiwan are surrounded by a hot and humid climate. Affected by global warming, heat waves are now becoming increasingly frequent and intense in Taiwan, which has led to an increasing number of heat-related illnesses, including heat cramps, heat exhaustion, and heat stroke. Among these illnesses, heat stroke is the most severe, which often occurs in a high temperature and calm weather.

As sports are becoming a more popular component of daily life, many wearable devices capable of detecting physiological information, automatically recording physical data, and tracking the user’s location have been developed to offer convenience and improve safety in sport activities. The improvement in safety is particularly important because the detection of physiological information enabled by these wearable devices can monitor the physical condition and predict potential safety risks for the user, thereby effectively reducing the possibility of getting injured in sports. Table 1 summarizes the pathological features and the corresponding physiological risk factors of heat stroke, obtained from relevant literature studies [2,3]. These findings can be used to increase the chance of early detection of heat stroke.

In their heat stroke prevention studies, Naoya Mizota et al. [4] proposed the concept of showing a heat stroke alert on users’ smartphones based on the environmental temperature—humidity index. Other studies have also suggested that the change in body temperature for individuals of different ages is also an indicator of potential heat stroke [5,6,7]. Many other wearable devices equipped with physiological information sensors can monitor changes in a patient’s condition using physiological information signals, such as electrocardiogram, electromyography, and electroencephalogram signals [8,9]. However, a simple device for assessing heat stroke risk based on pathological features and their physiological information that can be carried easily by individuals is still lacking. Such a device can advise the users to take appropriate precautions in case of potential heat stroke risk, and therefore, prevent heat injury.

Wearable devices have been used widely in everyday life with substantial impact on the way that we live. By integrating physiological sensors in wearable devices, the physiological information of an individual exercising (e.g., running) can now be monitored automatically and instantaneously. This information can be combined with recorded environmental conditions to predict the risk of heat stroke and further advise the user to take proper precautions. Because ordinary wearable devices have limited computational power, the physiological information collected by the sensors is usually first sent back to a paired smartphone through Bluetooth wireless communication and then processed by the smartphone [10]. Bluetooth wireless communication embedded in smart handheld devices has the advantage of low power consumption and easy connection [11]. While the operating range of Bluetooth communication is officially claimed to be 100 m, experiments have shown that the practical communication range is between 5 and 10 m [12]. Although such a distance can satisfy the requirements in most conditions [13,14,15,16,17,18], the ultimate distance between the sensor and the smart handheld device is still limited by the maximum available wireless communication distance if one wishes to monitor the physiological information of an outdoor runner instantaneously. For individuals performing outdoor sports activities, increasing the wireless communication distance of the wearable device can offer more convenience. A summary of the technical specifications of different wireless communication systems [19] is provided in Table 2. As shown in the table, LoRa is a promising wireless technology that can resolve the aforementioned issue owing to its advantageous transmission distance, transmission power consumption, and standby current. Specifically, it has a longer range of wireless signal transmission, a lower sensitivity, and a lower power consumption [12] than other wireless communication technologies. Therefore, it is an ideal candidate for use as a communication module in wearable devices.

In addition, fuzzy logic is different from the traditional binary logic, in which the state can be only described by 0 or 1. In fuzzy logic, a membership function with output values changing continuously between 0 and 1 is used to describe the state of a phenomenon [20]. Using binary logic to describe heat stroke can result in potential danger because heat injury has already occurred when a heat stroke is detected. Using fuzzy logic to describe the heat stroke can prevent potential heat injury based on the level of heat stroke derived by the fuzzy rule. Thus, fuzzy logic is suitable for use in heat stroke prevention.

In our previous work [21], we developed a wearable heat stroke detection device (WHDD) and demonstrated its heat stroke prediction capability for running. It should be noted that the usage of WHDDs is not limited to running alone. The WHDDs can be used to monitor body temperature and prevent the occurrence of heat stroke in any activity or exercise that carries the risk of heat stroke. In this study, we perform a more detailed analysis and experimental investigation from the perspective of information analysis and experimental subjects. Our results further demonstrate the superior applicability of the WHDD.

## 2. Materials and Methods

### 2.1. System Description

**Figure 1:**
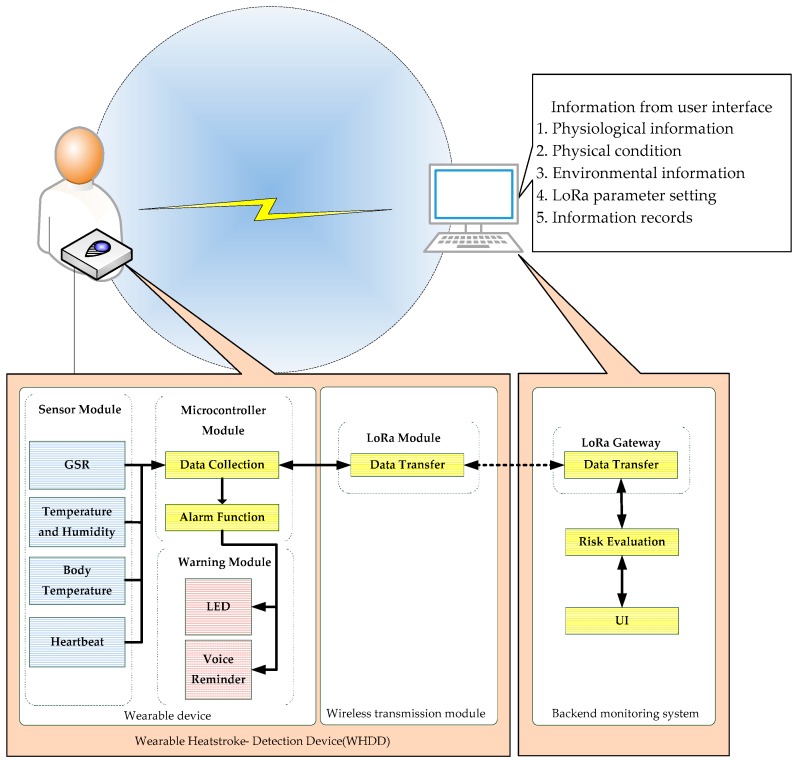
Architecture of the system.

**Figure 2:**
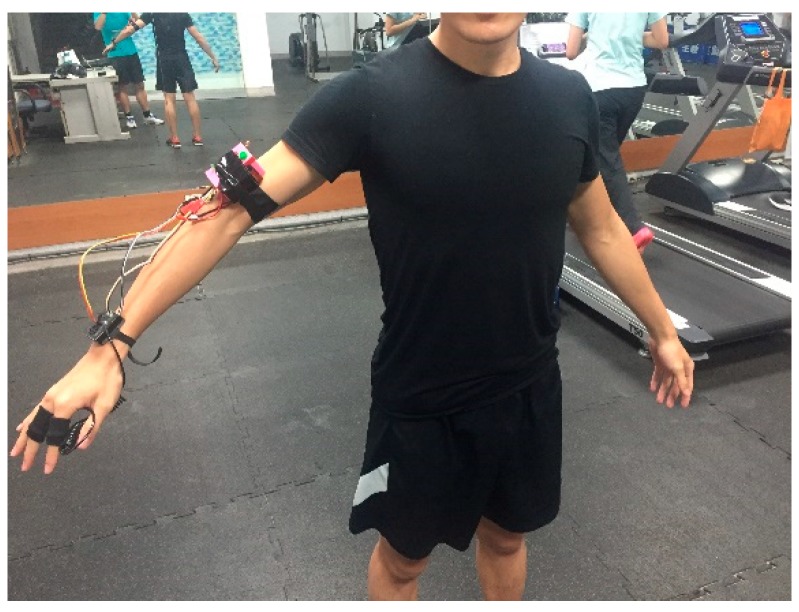
Photograph of the wearable heat stroke detection device (WHDD) on a human body.

As shown in Figure 1, the architecture of the WHDD comprises three main parts: a wearable device, a wireless transmission module, and a back-end monitoring system. The complete WHDD is shown in Figure 2. Detailed descriptions of the different components of the device can be found in our previous work [21].

#### 2.1.1. Wearable Device

The wearable device is further composed of three parts: the microcontroller, the sensor module, and the alert module. The microcontroller, based on an Arduino Nano board, is responsible for performing basic processing of the front-end data collected by the sensors, data filtering, and signal filtering. Subsequently, the microcontroller packs the data and passes them to the LoRa wireless transmission module, from where they are transmitted to the back-end monitoring system. The sensor module comprises four individual sensors that measure heart rate (World Famous Electronic), body temperature (MLX90614), environmental temperature and humidity (SHT75), and skin resistance (Grove-GSR) separately. The sensor module captures the main physiological information from the user’s body. This information is then transmitted to the back-end monitoring system by the wireless transmission module. Once the back-end system evaluates the risk of heatstroke from the received data, based on the risk level, the control buzzer warns the runner as follows: no alert means Safe situation, the LED turns on without the buzzer in Attention mode, the LED blinks and the buzzer beeps smoothly in Warning status, and the LED blinks and the buzzer beeps rapidly in Interdiction mode. The system suggests to the runner to ensure that appropriate measures are immediately taken in a dangerous situation to avoid heat stroke. The details of these procedures can be found in our previous work [21].

#### 2.1.2. Wireless Transmission Module

To achieve a large-distance, low-power, and low-cost design target, the LoRa module developed by iFrogLab is used in the wireless transmission module of our device. The LoRa transmission module enables transmission of the information collected by the sensors from the wearable device to a terminal device. The wireless transmission module employs universal asynchronous receiver/transmitter (UART) for signal communication. Control of the data transmission is achieved using the attention (AT) command system. These approaches facilitate the integration of different components to construct the device.

#### 2.1.3. Back-End Monitoring System

After collecting and transmitting the physiological information using the sensors and LoRa, respectively, this information is received by the back-end monitoring system, where the heat stroke risk is analyzed. The primary functions of the back-end monitoring system include recording the user’s physiological information, physical status, and environmental information, as well as setting the parameters for LoRa.

### 2.2. Planning and Design of Heat Stroke Detection Workflow

**Figure 3:**
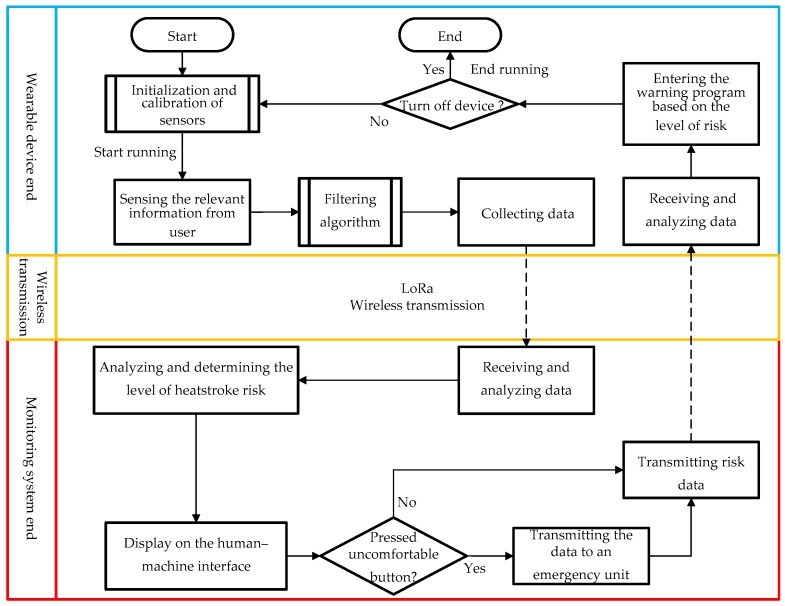
Workflow of the WHDD.

After introducing the system architecture of the WHDD, we define the desired operation of the device as well as the associated techniques and hardware required. In this section, we discuss the design of the workflow for heat stroke detection and for the specific functions associated with each of the three architectures (the wearable device, the wireless transmission module, and the back-end monitoring system). Figure 3 shows the workflow of the entire system.

#### 2.2.1. Wearable Device

This section describes the workflow for the target function of the WHDD. The overall workflow can be divided into two stages, namely, the stage before running and during running. The workflow for each section is described in detail below.

**Figure 4:**
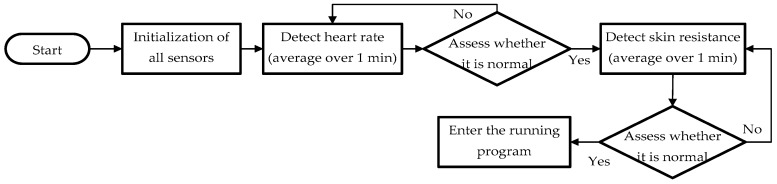
Workflow for collecting physiological information before running.

During the first stage (before running), the physiological information of the user is recorded. This information will be compared with the physiological information of the user during running. The change in the physiological information before and during running is an important factor for predicting heat stroke risk. Two physiological features, the heart rate and the skin resistance, of the user are recorded by the sensors during this stage. The overall workflow is shown in Figure 4.

This workflow comprises the following steps:

1. Initialization of all the sensors.
2. The heart rate sensor monitors the user’s heart rate for 1 min with a sampling frequency of 2 Hz (one sample every 0.5 s). After 1 min, a total of 120 measurement points are obtained, whose average value is considered as the user’s heart rate before running.
3. Determine whether the heart rate is normal. The heart rate of a normal adult ranges from 50 to 90 bpm [22]. If the heart rate cannot be detected by the sensor, equals zero, or falls within the range associated with an adult in a non-resting condition, then an abnormal phenomenon is identified, and the heart rate is measured again.
4. The user’s skin resistance is measured through the galvanic skin response for 1 min with a sampling frequency of 2 Hz (one sample every 0.5 s). A total of 120 measurement points are obtained and the average value is used as the user’s skin resistance before running.
5. Determine whether the user’s skin resistance falls within the normal range. The skin resistance of an adult under resting conditions is approximately 10–50 μS [23]. If the skin resistance cannot be measured, equals zero, or falls within the range associated with an adult in non-resting conditions, then an abnormal phenomenon is identified and the skin resistance is measured again.

**Figure 5:**
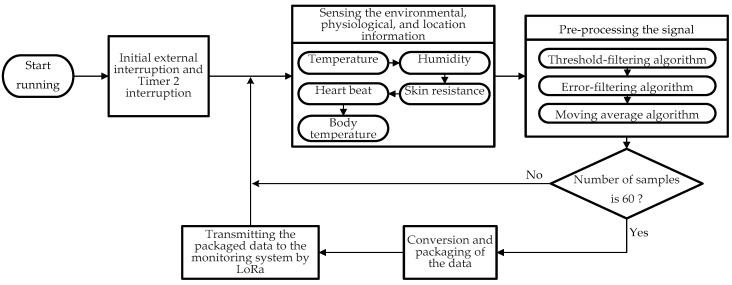
Workflow for extracting information on the surrounding environment and the user’s physiological condition (main program).

In the second stage (during running), the wearable device mainly captures information about the surrounding environment and the user’s physiological condition. Subsequently, this information is packaged and transmitted to the monitoring end by the LoRa wireless transmission module. The main workflow is shown in Figure 5.

This workflow comprises the following steps:

1. Initial timer interruption program and external interruption program. The timer interruption program allows the wearable device to receive the heat stroke risk level from the monitoring system on a regular basis. The external interruption program enables the user to ask for help by pressing a button on the device. In this case, the device immediately sends the physiological and environmental information back to the monitoring end.
2. Determine whether the device must be turned off, namely, by removing the battery from the device and shutting down the program.
3. Collection of the user’s physiological and environmental information by the sensors (e.g., temperature and humidity sensor). The information is collected in the following order: environmental temperature and humidity, skin resistance, heart rate, and body temperature. This information is collected at intervals of 3 ms. Therefore, 15 ms are required to complete an entire cycle of information collection.
4. The heart rate and body temperature signals are preprocessed, first by a threshold-filtering algorithm, then by an error-filtering algorithm, and finally by a moving-average algorithm. When the number of samples reaches 60, all the filtered information is packaged and transmitted to the monitoring system by LoRa. The interval between each data transmission is approximately 15 ms × 60 = 900 ms (approximately 1 s)
5. When the number of samples is less than 60 or the data transmission is finished, the device continues executing the workflow from step 2.

The detailed signal processing workflows for the two most important indices for evaluating the heat stroke risk, i.e., the heart rate and body temperature, are discussed below.

Because the heart rate sensor is designed to be positioned on the user’s wrist, the movement of the arms while running can potentially induce errors in the heart rate measurements. To resolve this issue, a threshold-filtering, error-filtering, and moving-average strategy is adopted to preprocess the heart rate signal and mitigate measurement errors. The code of the program is shown in Algorithm 1.

**Algorithm 1** Heart rate filtering
1:Global Variables:2:    *TrueHR*                                     △*TrueHR* is the final filtered value of user’s heart rate3:    *count* = 0, *mincount* = 0, *maxcount* = 04:    Let *HR*[60] to be a new array5:    *HR*[0] = *RelaxHR*                                                        △*RelaxHR* is the user’s relax heart rate 6:    **function** *HRThresholdFilter*(*Value*)                                          △*Value* comes from HR sensor 7:        *count* + +8:        **if** 50 ≦ *value* ≦ 190 **then**9:            *HR*[*count*] = *Value*10:        **else**11:            *HR*[*count*] = *HR*[*count* − 1]12:        **end if**13:        Call function *HRErrorFilter*(*HR*[*count*])14:        **if** *count* = 59 **then**15:            Call function HRMovingAverage()16:        **end if**17:    **end function**18:    **function** *HRErrorFilter*(*Value*) 19:        *Error* = *Value* − *HR*[*count* − 1] 20:        **if** *Error* < −10 **then**21:            *mincount* + +22:            **if** *mincount* ≦ 6 **then**23:                *HR*[*count*] = *Value* − Round(*Error* ∗ 1.05)24:            **else**25:                *HR*[*count*] = *HR*[*count* − 1]26:                *mincount* = 027:            **end if**28:        **else if** *Error* > 25 **then**29:            *maxcount* + +30:            **if** ( *thenmaxcount* ≦ 2)31:                *HR*[*count*] = *Value* − Round(*Error* × 0.6)32:            **else**33:                *HR[count] = HR[count − 1]*34:                *maxcount* = 035:            **end if**36:        **else**37:            Keep *HR*[count]38:        **end if**39:    **end function**


In the heart rate threshold-filtering program, heart rate signals falling outside the range of 50 to 190 bpm are filtered out. This is because the heart rate of an adult at rest ranges between 50 and 90 bpm [22]. Therefore, 50 bpm is selected as the lower limit of the heart rate. The upper limit is selected based on the study by Tanaka et al. [24], who proposed in 2001 that the maximum heart rate ($HR_{Max}$) of an individual during exercise should be calculated based on the age instead of the gender. Roy et al. [25] in 2015 reviewed all the existing equations for calculating the maximum heart rate during exercise. They found that the equation proposed by Tanaka is rather accurate. This equation is given by

(1) $$HR_{Max}=208-0.7\times age$$

Here, we assume the age to be 26 and derive the maximum heart rate to be approximately 190. Therefore, the upper limit of the heart rate is selected to be 190.

In the heart rate error-filtering program, each measured heart rate ($HR_{t}$) is compared with the heart rate recorded in the previous cycle. The error between neighboring measurements is given by

(2) $$Error=HR_{t}-HR_{t-1}$$

This difference is used to revise the heart rate measurement based on a comparison between the performance of the heart rate sensor used in this study and that of a commercial heart rate belt. A fixed equation is derived to convert the measured heart rate to the actual value based on the difference between neighboring heart rate measurements. The revised heart rate is given by the following equation

(3) $$\{\begin{array}{c} HR_{fix}=HR_{t}-Error\times 1.05,\;\;\;\;Error<-10\\ HR_{fix}=HR_{t}-Error\times 0.6,\;\;\;\;\;\;Error>25\; \end{array}$$

Finally, when the number of heart rate measurements reaches 60, a heart rate moving-average program is executed to obtain the average value of the heart rate measurements obtained over 60 cycles. The final value is used as the anticipated heart rate ($HR_{True}$), which is given by

(4) $$HR_{True}=\frac{\sum_{0}^{t}HR_{t}}{60},\;t=1\ldots 60$$

The sensor used for measuring body temperature in this study is a non-intrusive sensor. First, it measures the human skin temperature using an infrared sensor and then it converts that to the adult body temperature using a conversion equation. This temperature sensor is placed on the inner side of the user’s wrist. Owing to the low thickness of the skin, this location is the most suitable place for measuring body temperature. According to a study by John Gammel [26], the following equation, together with the parameters (*α*) listed in Table 3, are used to convert skin temperature to core temperature.

(5) $$T_{Core}=T_{Skin}+\alpha \times \left(T_{Skin}-T_{Ambient}\right)$$

However, we found that the sweat generated during exercise reduces the surface temperature of the skin, and therefore, results in a lower body temperature. This impact also varies significantly with different levels of sweating for different people. Specifically, the measured skin temperature is increasingly smaller than the actual skin temperature for those generating a larger amount of sweat during exercise and vice versa. Therefore, the error of the core temperature obtained using the conversion equation is greater with increasing exercise time. To reduce the error induced by this physical phenomenon, the temperature data are filtered and processed using the program code shown in Algorithm 2.

**Algorithm 2** Body Temperature filtering
1:Global Variables:2:*BodyTemp*                 △*BodyTemp* is the final filtered value of user’s body temperature3:*TempDiff* = 04:Let *Temp*[60] to be a new array5:**function***TempThresholdFilter*(*Value*)                 △*Value* comes from MLX90614 sensor6:        **if** 28 <= *Value* <= 35 **then**7:            *Temp* = 35 − *Value*8:            **if** *Temp* ! = *TempDiff* **then**9:                *TempDiff* = *Temp*10:                *Temp*[*count*] = *Value* + *TempDiff* × 1.511:            **else**12:                *Temp*[*count*] = *Temp*[*count* − 1]13:            **end** if14:        **else if** 35 < *Value* < 40 **then**15:            *Temp*[*count*] = *Value*16:        **else**17:            *Temp*[*count*] = 3618:        **end if**19:        **if** *count* = 59 **then**20:            Call function *TempMovingAverage*()21:        **end if**22:
**end function**



After converting the skin temperature to the core temperature ($T_{Core}$), the core temperature is processed by a temperature-threshold filter to yield a reasonable body temperature. This revision is based on a comparison between the body temperature measured using the system developed in this study and the value measured using an ear thermometer. A compensation equation is derived from this comparison to revise the core temperature measured by our device. The equation and the applicable temperature ranges are given by

(6) $$\{\begin{array}{c} T_{Fix}=T_{Core}+\left(35-T_{Core}\right)\times 1.5,\;\;\;31\leq \;T_{Core}\leq 35\\ T_{Fix}=T_{Core},\;\;\;\;\;35<T_{Core}<40\;\\ T_{Fix}=36,\;\;\;\;\;else\;abnormal\;condition \end{array}$$

When the measured core temperature ($T_{Core}$) is in the range 31–35 °C, the real body temperature is obtained by compensating the difference between the core temperature and 35 °C proportionally. When the measured core temperature is 35–40 °C, no further revision is required. If the measured core temperature is outside the above ranges, it is assigned with a constant value of 36.0 °C, as explained below. According to Reference [27], humans are warm-blooded animals, and the normal body temperature (no disease) of an adult human range between 35.0 and 37.0 °C based on forehead temperature measured by an infrared temperature gun. Therefore, all abnormal body temperatures were converted to the average human body temperature of 36.0 °C in this study.

Finally, similar to the process used for the heart rate and skin resistance signals, the body temperature data are also processed by a moving-average program that calculates the average value of 60 temperature measurements. Equations (3) and (4) are used to yield the final body temperature of the user ($T_{Body}$).

#### 2.2.2. Monitoring System End

After the user’s information is transmitted from the wearable device to the monitoring system, it is imported by the monitoring system to the fuzzy controller designed in this study. The physiological information measured by the sensors and collected by the microcontroller, such as skin resistance, safety factor, human body temperature, and heart rates, is used as input variables for performing fuzzy inference based on a fuzzy rule database, after these input variables are fuzzified. The final results are defuzzified to yield the instantaneous risk level of the user automatically. The details of this process can be found in our previous work [21]. Additionally, a human–machine user interface (UI) was developed by combining the back-end monitoring system with a C# program. This UI is used to display the physiological information of the user.

### 2.3. Experiment

This section discusses the experimental process and compares data measured in static conditions (before exercising) and dynamic conditions (during exercise) to confirm the applicability of the WHDD.

#### 2.3.1. Static Experiment

Heart rate and body temperature are the two most important indices for detecting heat stroke [28,29]. To validate the accuracy of these two indices, as measured using the proposed device, we performed a 90-s static experiment. The original heart rate and body temperature measured by the sensors were compared with the results obtained after applying the numerical-filtering algorithm and the conversion formula proposed in this paper. Such a comparison allows us to evaluate the stability of the sensor and the performance of the filtering algorithm. Additionally, the values recorded by the heart rate and body temperature sensors every 10 s were also compared with measurements obtained with existing commercial products to verify the accuracy of our device. Specifically, the CK-102S [30] instrument, purchased from CHANG KUN, was used to measure the heart rate with a ±5% accuracy. The UE-0042 [31] instrument, purchased from nac nac, was used to measure the ear temperature with a ±0.2% accuracy. The deviation between the raw values detected by each sensor and those obtained from the commercial instruments were explored by experiments.

#### 2.3.2. Dynamic Experiment

Four adults between the ages of 25–37 participated in the dynamic experiment, as shown in Table 4. The participants were required to wear the WHDDs and run on a treadmill for 15 min in an indoor environment with a temperature of 28.9 °C and a humidity of 68.2%. The participants performed the exercise at different intensities. The entire test comprised three stages, including warm-up (running at 8 km/h for 10 min), accelerating (running at 10 km/h for 2 min), and intense exercise (running at 12 km/h for 3 min). Increases in running intensities will increase the discomfort of the runners, but the amount of discomfort felt by each individual will be different because they have different levels of fitness. Therefore, the users could press the button on the device to send feedback when they felt uncomfortable while running. This feedback was used for experimental data analysis and validation.

**Figure 6:**
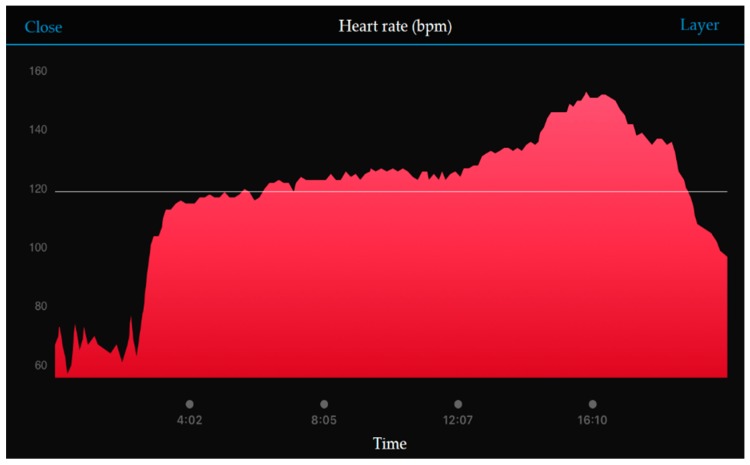
Heart rate data measured with the commercial heart rate belt.

**Figure 7:**
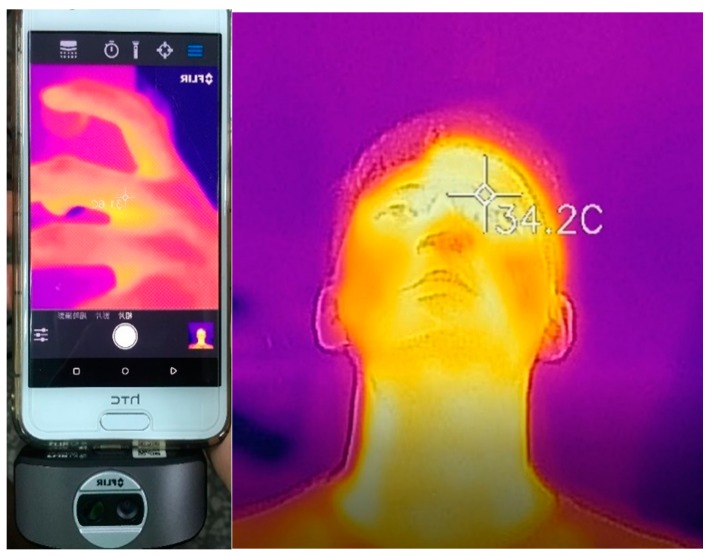
Thermal camera and measured dynamic body temperature map.

Additionally, when the user was running, the movement of the arm caused the wearable device to loosen, which resulted in errors in the sensor measurements. Although such a scenario was inevitable, the filtering algorithm could detect and remove these abnormal signals. Particularly, because the heart rate and body temperature were measured by non-intrusive methods, their values suffered from the greatest errors. Furthermore, because the heart rate and temperature sensors were only fixed on the skin surface, they could be affected substantially by the user’s motion. Therefore, the commercial product HRM-Ru [32] was used to obtain the heart rate under exercise conditions, as shown in Figure 6. A FLIR ONE [33] thermal camera was used together with a smartphone application to obtain the instantaneous body temperature, as shown in Figure 7. These measurements were compared with those obtained by the WHDD.

To probe the effectiveness of the WHDD in outdoor exercises, an outdoor running test was conducted with five adults in the evening. The test environment was a standard 400 m track, and the ambient temperature and humidity were 23.5 °C and 80%, respectively, which is equivalent to an environmental danger coefficient of 31.5. The test was carried out by having the test subjects run five continuous laps (2 km) around this track within their individual limits. The physiological differences between the test subjects are shown in Table 5.

## 3. Results

### 3.1. Static Experiment

#### 3.1.1. Heart Rate

**Figure 8:**
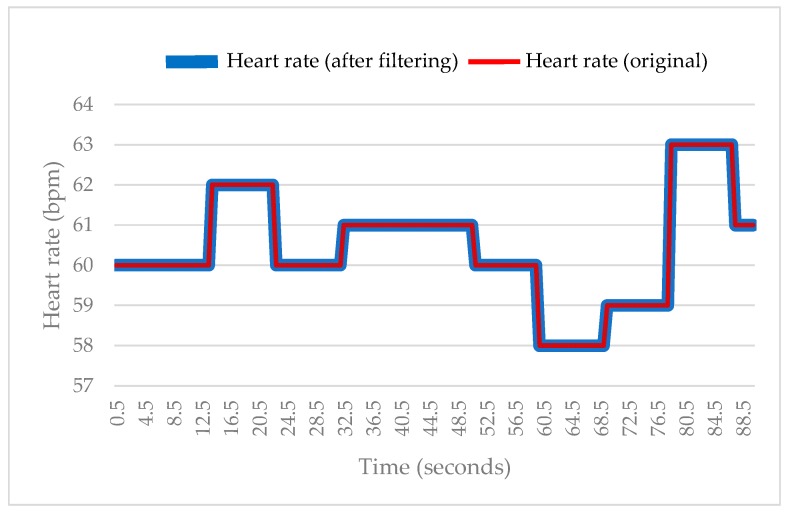
Comparison between original and filtered heart rate when the user was at rest.

As shown in Figure 8, all the original heart rate values fall within the normal range (50–90 bpm) [22] when the user is at rest. The data obtained after filtering by the microcontroller were found to overlap with the original data. This finding suggests that the sensor can measure the heart rate accurately when the user is at rest. Additionally, no significant measurement fluctuation was observed during the test and there was almost no difference between the original data and the filtered data.

Additionally, the average heart rate measured using the commercial wrist sphygmomanometer was approximately 56 bpm. The differences between the data measured using the commercial instrument and the data measured using the WHDD after filtering are summarized in Table 6. These results show that the average difference between the measurement obtained using the commercially available sphygmomanometer and the device developed in this study is approximately 0.1. Therefore, the sensor integrated in the WHDD can be used to measure the heart rate.

#### 3.1.2. Body Temperature

**Figure 9:**
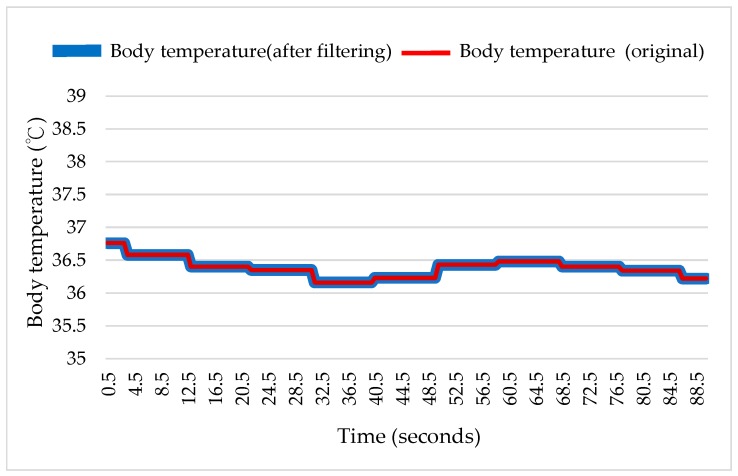
Comparison between the original and filtered body temperature when the user was at rest.

As shown in Figure 9, no significant difference was observed between the original data measured by the sensor and the filtered data when the user was at rest. This is primarily because all of the body temperatures obtained after conversion are within a reasonable range (between 35 °C and 40 °C), with an average value of approximately 36.5 °C. Additionally, we see in the figure that the sensor measurement is rather stable when the user is at rest. The maximum error was less than 1 °C, which confirms that the sensor integrated in our device can be used to measure body temperature.

Next, a commercial infrared ear thermometer was used to measure the body temperature when the user was at rest. The average body temperature measured by the commercial instrument varied by 0.14 °C from the temperature measured by the proposed device. The error comparison is shown in Table 7.

### 3.2. Dynamic Experiment

#### 3.2.1. Heart Rate and Body Temperature

The heart rate and body temperature exhibited the greatest errors among all the physiological information indices by the sensors. Therefore, the dynamic experiment was focused on investigating these two indices. The heart rates measured by the WHDD during running were compared with those measured using a commercially available commercial heart rate belt and the associated smartphone application. This comparison is shown in Table 8.

Next, the dynamic temperature data obtained during running were compared with the instantaneous body temperature measured using a commercial thermal camera in combination with a smartphone application, as shown in Table 9.

#### 3.2.2. Heat Stroke Risk Indicator

**Figure 10:**
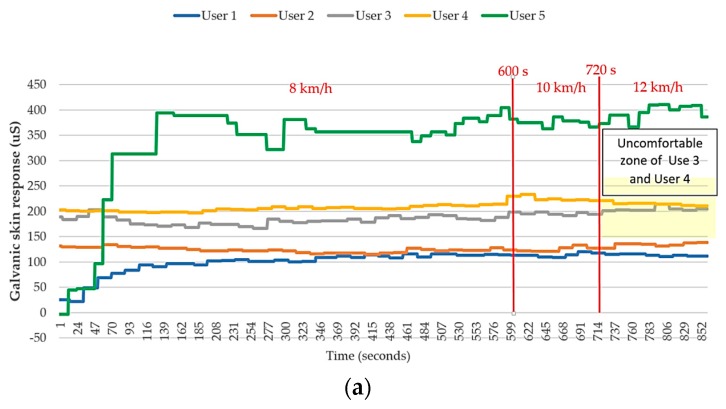

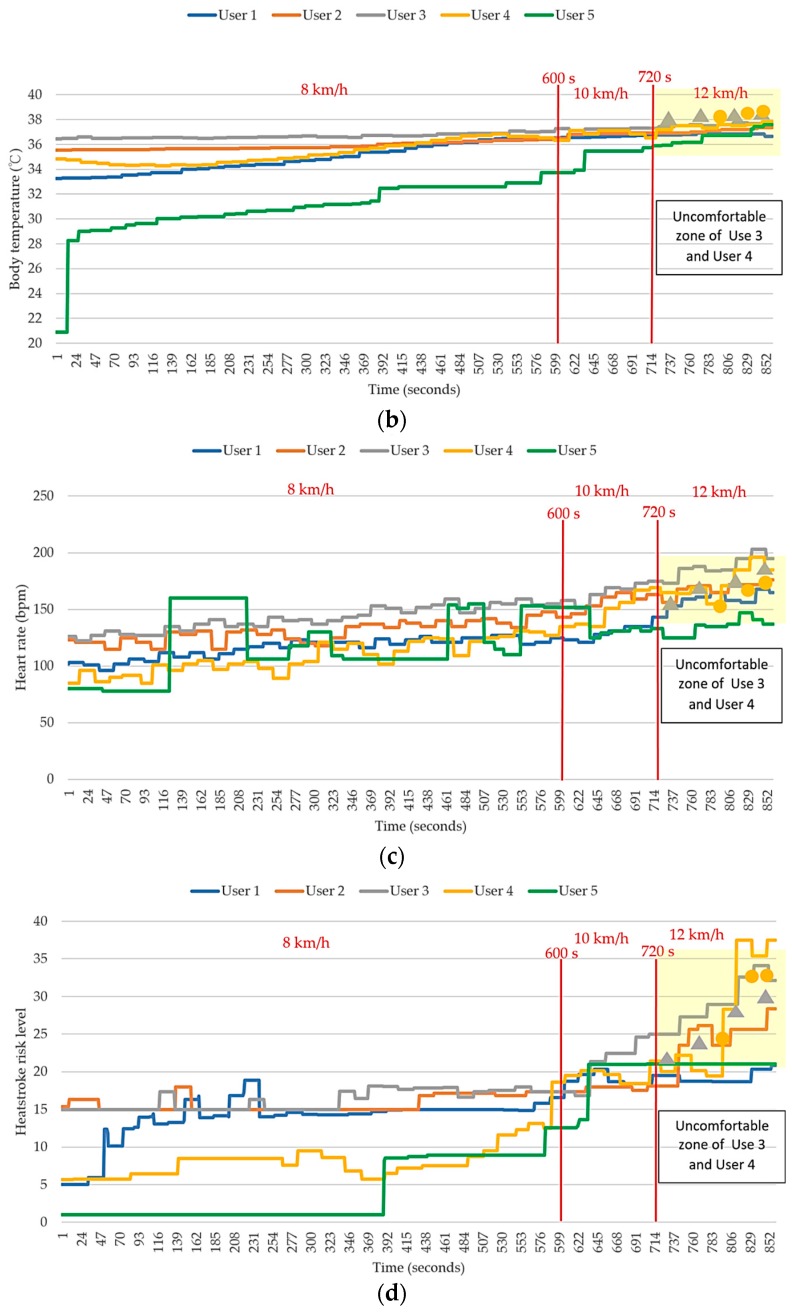
Comparison between the physiological data and the feedback signals of the four users during running. (**a**) Galvanic skin response (GSR); (**b**) body temperature; (**c**) heartbeat; and (**d**) heatstroke risk level.

Figure 10 shows a comparison between the physiological data and the feedback signals of the four users during running. Here, user 5 and user 1 are the same participant. Because the change in environmental temperature and humidity were negligible and all of the users exercised under suitable temperature and humidity conditions, the relationship between the environmental temperature/humidity and heat stroke risk are not discussed in this paper.

First, we found that the data associated with user 5 was very different from those associated with the other four users because they did not undergo the filtering process developed in this study. Instead, these data were extracted directly from the raw measurements to predict the heat stroke risk level. Particularly, the body temperature and heart rate of user 5 varied more significantly from those of the other four users. Although the data still exhibited a reasonable trend, in accordance with the model of an individual performing exercise, the large data fluctuations in a continuous time period resulted in large fluctuations in the heat stroke risk indicator. Therefore, very different heat stroke risk predictions are provided by the device in a short time. Such a high instability issue could cause the user and the system to make wrong assessments. In contrast, the data associated with the other four users were very stable. Therefore, the heat stroke risk levels were also found to be stable for these users.

**Figure 11:**
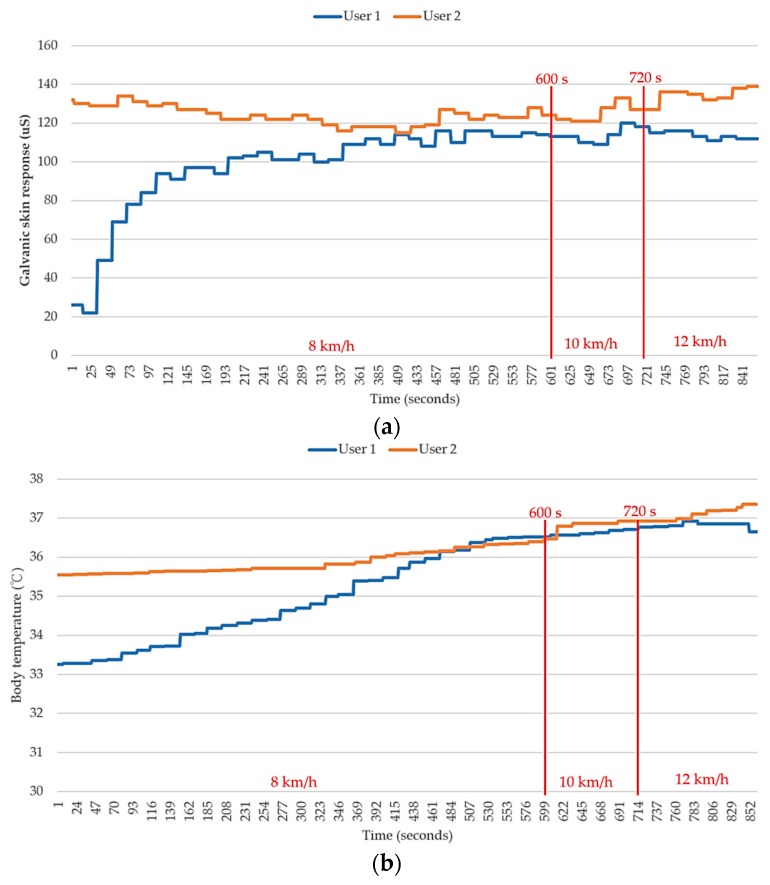

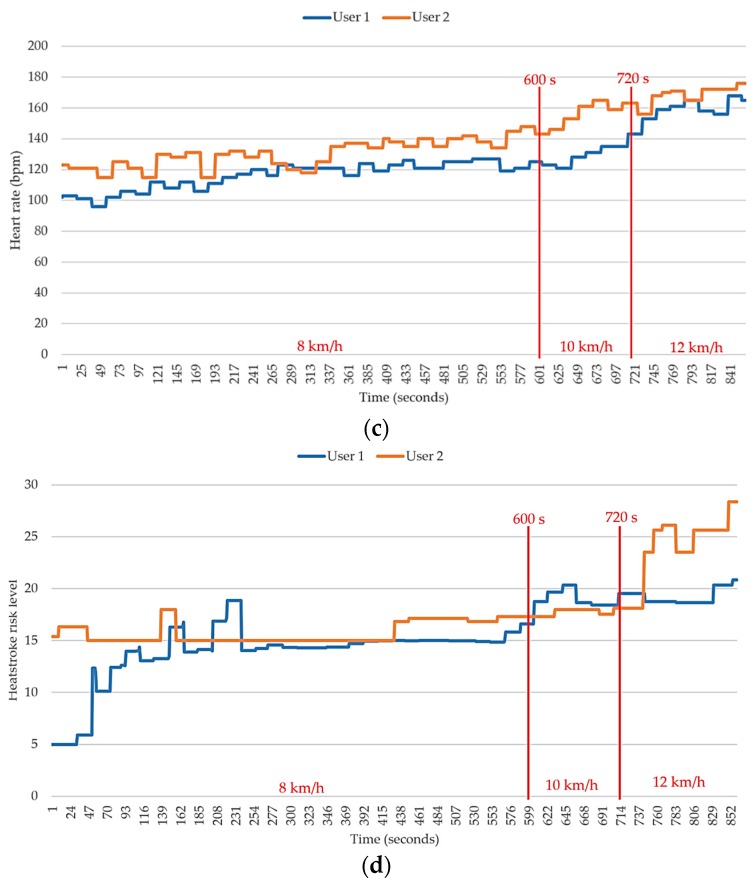
Comparison of skin inductance values in the testing group that did not report uncomfortable conditions (user 1 and 2). (**a**) Galvanic skin response (GSR); (**b**) body temperature; (**c**) heartbeat; and (**d**) heatstroke risk level.

**Figure 12:**
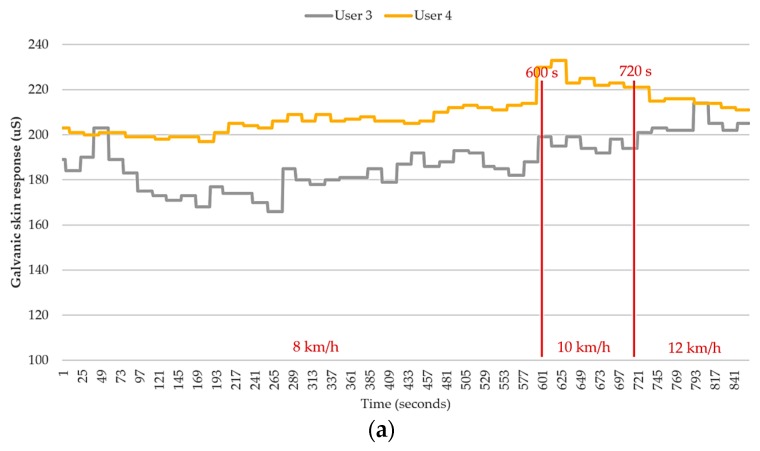

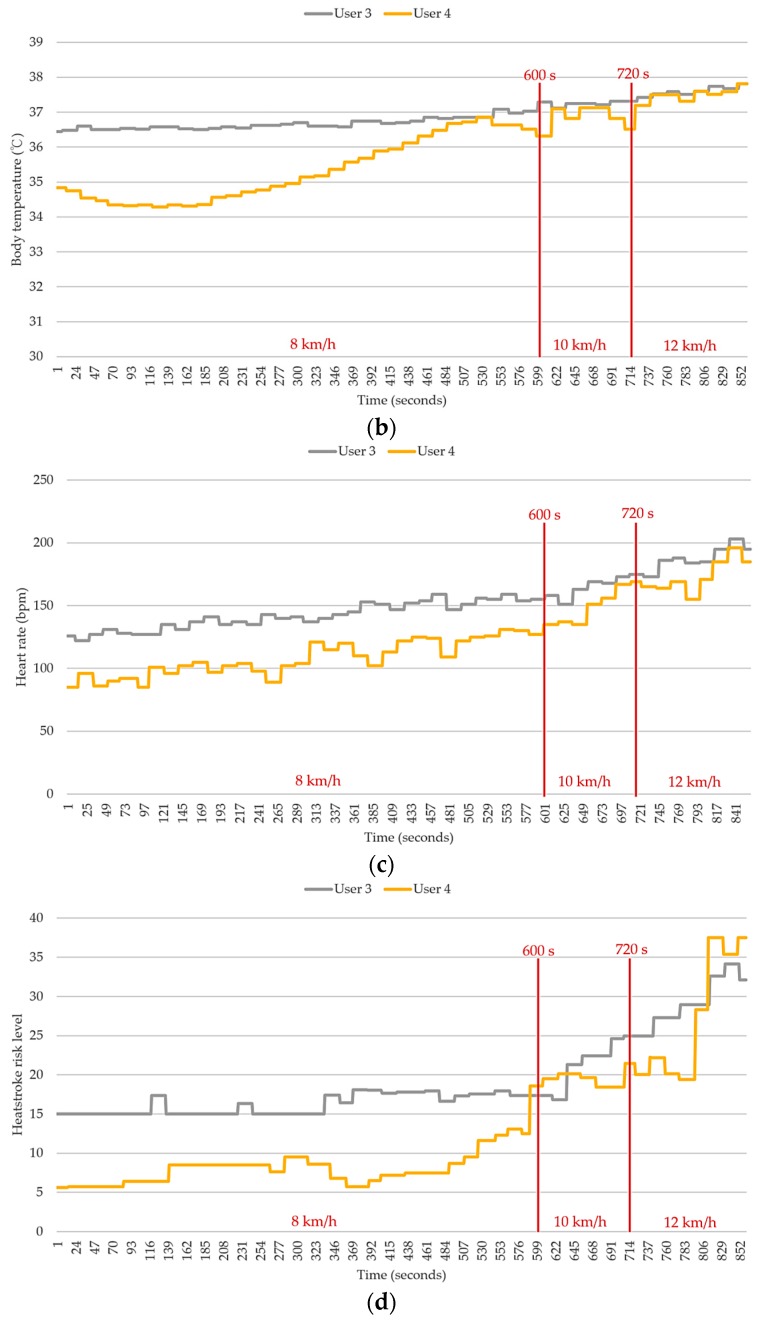
Comparison of skin inductance values in the testing group that reported uncomfortable conditions (user 3 and 4). (**a**) Galvanic skin response (GSR); (**b**) body temperature; (**c**) heartbeat; and (**d**) heatstroke risk level.

Next, a detailed analysis was performed on the conditions of the remaining four users. As shown in Figure 11 and Figure 12, both user 3 and user 4 provided “uncomfortable” feedback to the system, while user 1 and user 2 did not provide any uncomfortable feedback during the exercise. The results were divided into two groups based on the feature of “uncomfortable” and analyzed by comparing the numerical values. With respect to the change in skin inductance (skin resistance), both data groups showed a stable condition in the measurements. This finding indicates that all four users experienced continuous sweating while running. Therefore, the skin resistance changed accordingly during the process. However, the skin inductance of individual was determined by the reference value of the static skin resistance. In other words, the skin resistance is always different for different individuals. Therefore, we infer that the occurrence of uncomfortable conditions in this group was apparently not caused by the lack of sweating but by other physiological factors.

**Figure 13:**
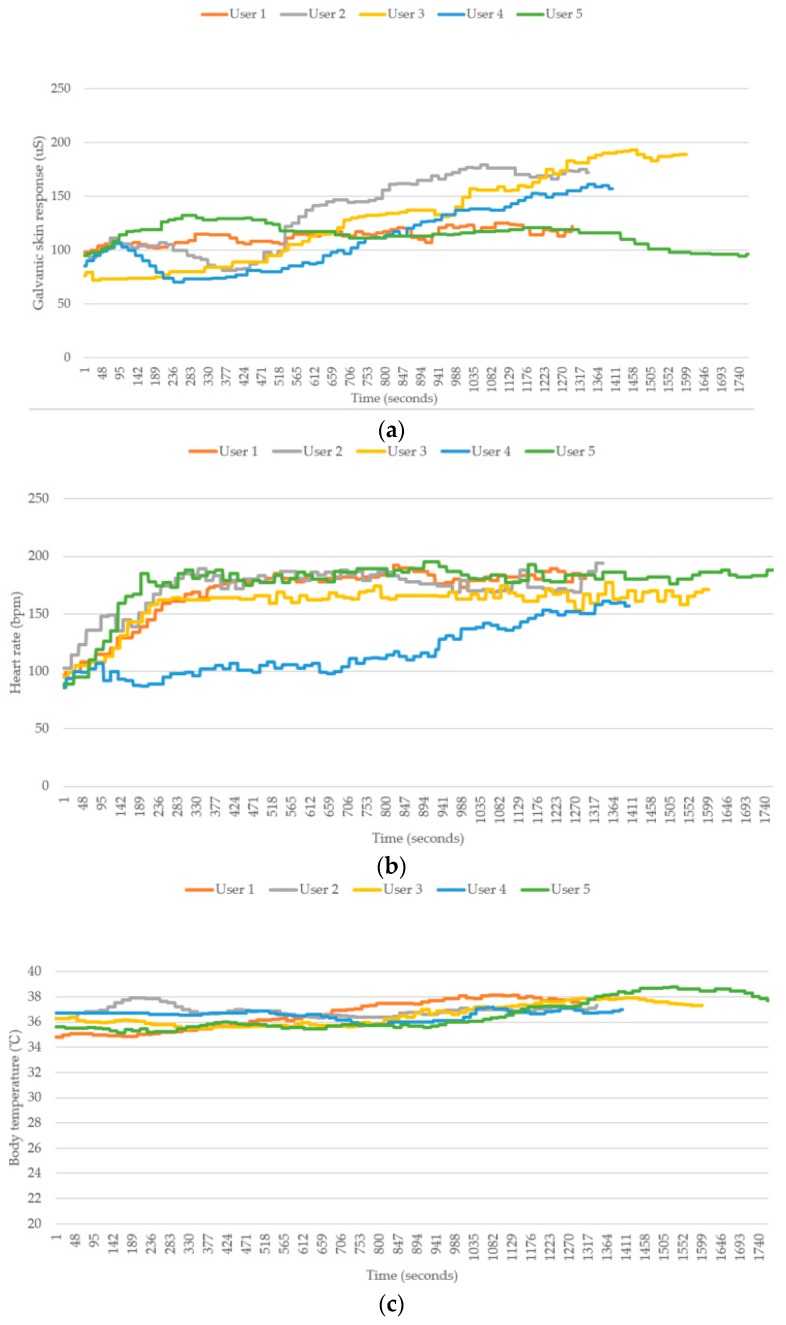

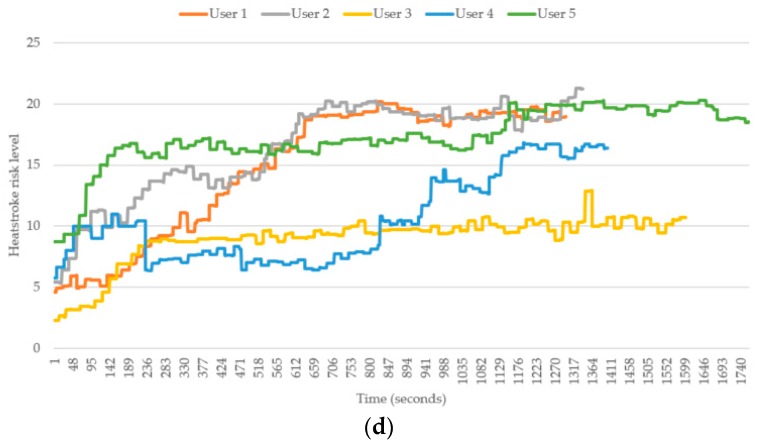
Comparison between the physiological data and the feedback signals of the five users during running outdoors. (**a**) Galvanic skin response (GSR); (**b**) body temperature; (**c**) heartbeat; and (**d**) heatstroke risk level.

Figure 13 shows the results of the outdoor WHDD experiment. Because the physical condition of each test subject was different, the durations in which they completed the 2-km run were all different. User 1, who exercises regularly, completed the 2-km run in the shortest amount of time, whereas User 5, who had the worst physical condition, required the longest duration of time to complete the run. Furthermore, because the environmental danger coefficient of this experiment was lower than that of the indoor experiment (31.5 versus 35.72), it was observed that the risk of heat stroke in this low-risk environment, as evaluated by the WHDD system, was generally lower than that of the indoor experiment.

## 4. Discussion

The rise of body temperature is a common phenomenon for humans when running. Additionally, the highest temperature of all four users never reached a dangerous level (40 °C). This finding suggests that the central control of the human body functions properly to maintain a normal body temperature. However, close examination reveals that the body temperatures of the users in the first group were higher than those in the second group. Although their body temperatures were still within a normal range, a high body temperature can still significantly increase the risk of heat stroke in the calculation. Nevertheless, no substantial difference in body temperature was observed between different users. Therefore, the body temperature is unlikely to be the factor causing an uncomfortable feeling. However, a reasonable guess is that if all four users keep running continuously, there is a high possibility that the body temperature of some users will eventually exceed 40 °C. This could result in a significant increase in heat stroke risk and to a dangerous situation.

The last physiological factor, the “individual heart beat,” is presumed to be the main factor causing an uncomfortable feeling. It can be seen in the Figure 10 that the conditions of all four users are rather normal during the initial stage—particularly, in the first 10 min, when the users were jogging at 8 km/h. When the users started running at 10 km/h, a significant increase in heart rate was observed for users 2, 3, and 4. This phenomenon is consistent with the physiological changes that occur in the human body when performing exercise. No uncomfortable condition was observed during this stage, which indicates that the heart rate of each user was within a reasonable range associated with exercise. When the users started running at 12 km/h, the heart rate of user 3 was found to increase greatly and to be considerably higher than that of the other three users. Next, an uncomfortable signal was sent from user 4 when the total exercise time approached 800 s. Afterwards, the heart rate of user 4 increased suddenly as well. Although the increased heart rate of user 4 was still lower than that of user 3, it was still much higher than those of the other two users. Although the heart rate of user 2 was also rather high, it only increased slightly and remained mostly stable during the stage in which the users were running at 12 km/h. This indicates that the high heart rate associated with the exercise load was still acceptable for the user.

By combining the physiological information and the feedback signals of each user, three conclusions can be drawn from the study:

First, from the perspective of individual data, it can be seen that the heat stroke risk indicator of the users who gave uncomfortable feedback falls within the alert (21–30) and dangerous (31–40) zone. For the users that did not give uncomfortable feedback, however, the highest heat stroke risk indicator falls only within the alert zone (21–30). Therefore, we conclude that the heat stroke risk indicator obtained by the fuzzy controller can be used as a reference for predicting the danger of heat stroke. However, the actual body condition of an individual with a heat stroke risk indicator in a fuzzy area (e.g., the alert zone) can only be known by questioning the person. Only by doing so can we determine the possibility of heat stroke for this individual.

Second, from the perspective of individual data, the physiological factor and the actual physiological reaction of each individual are found to be in good agreement. We can infer with confidence that the heart rate is the major reason causing the uncomfortable feeling to the user. A second factor is the body temperature, which incurs a reaction slightly later than the reaction induced by the heart rate. This is because the rise of body temperature is a normal phenomenon for humans performing exercise. A body temperature reaching 39 °C can still be considered as normal. However, if the individual keeps running with a high heart rate (high load), the body temperature will inevitably rise to a dangerous level. The last factor related to heat stroke is the skin resistance. The reaction induced by skin resistance occurs even later than that induced by body temperature. This is because the human body must dissipate excessive heat by sweating to maintain a constant temperature. The skin resistance starts changing significantly (decrease from large to small, accompanied by a reduction in sweat) only when the body temperature becomes too high. This scenario indicates a shift from normal sweating to a no-sweating condition. Therefore, the heat cannot be dissipated effectively from the human body. At this stage, the user is most likely already affected by heat stroke. Therefore, it is necessary to use the WHDD to predict the possibility of heat stroke and the associated uncomfortable symptoms for a particular user. In this case, an appropriate reminder can be provided to the user to avoid suffering a heat stroke.

**Figure 14:**
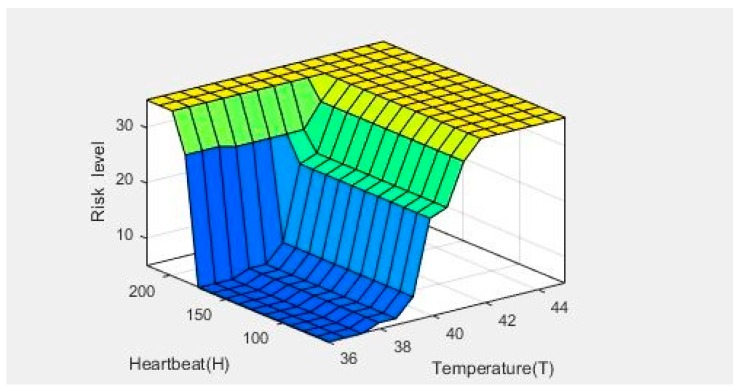
Three-dimensional relationship map between heart rate, temperature, and heat stroke risk level.

A systematic assessment of the relationship between the heart rate, body temperature, and heat stroke risk value was performed. The heat stroke risk value was calculated by the fuzzy controller. Fuzzy theory is mainly based on expert systems—i.e., the experience of the user—whereas the fuzzy rules are obtained from the literature, users’ feedback, and repetitive tests. In the previous section, an analysis of the numerical values obtained from the actual experiments was performed based on Figure 10. From this analysis, we can obtain the order of reaction to each physiological factor associated with heat stroke, which is: heart rate > body temperature > skin resistance. Therefore, a similar result is expected when analyzing the relationship between heart rate and temperature with heat stroke risk using the heat stroke fuzzy controller designed in this study. Figure 14 shows the relationship between these three factors, obtained by analyzing the results in MATLAB, as the figure clearly shows that the slope associated with the relationship between heart rate and heat stroke is much larger than that associated with the relationship between body temperature and heat stroke risk. This finding also confirms that the device developed in this study can truly reflect the possibility of suffering heat stroke during exercise for an individual with certain physiological characteristics.

**Figure 15:**
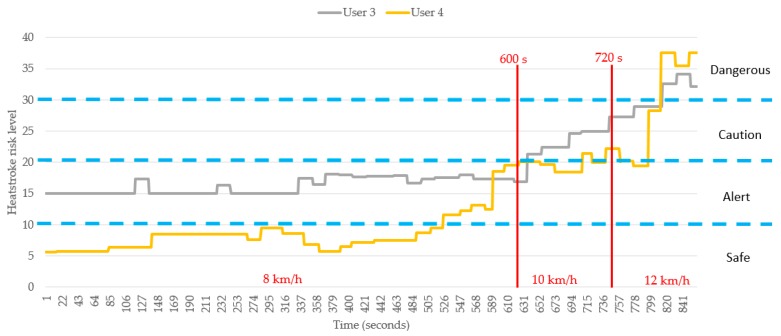
Comparison of heat stroke risk indicators for users feeling uncomfortable during the experimental tests (users 3 and 4).

Subsequently, the feedback of the users who reported an uncomfortable feeling was compared and validated against the predictions of our system. The analysis of the comparison shown in Figure 15 yields the results shown in Table 10 and Table 11. Based on the data of user 3, the system indicates a “dangerous” condition after 816 s. This prediction is 84 s (1 min 24 s) later than the first feedback provided from the user (732 s). For user 4, however, the system indicates a “dangerous” condition starting at 814 s. This prediction is only 2 s earlier than the first feedback provided by user 4 (816 s). Based on the analysis results on these two individuals, we can first conclude that the heat stroke detection function of our system can be affected by the physiological differences between different individuals and their distinct exercising habits. However, the system developed in this study is capable of detecting potential heat strokes. If the time factor is excluded, our system can effectively reflect the physiological condition of a user, predict the possibility of suffering heat stroke, and assist in cases of danger. To resolve the issue of the differences between different individuals, we can modify the parameters of the fuzzy controller according to the characteristics of the individual. Thus, the system can be revised to better match the condition of a particular individual. In the future, we expect to introduce the concept of machine learning to our system, which can automatically correct the associated parameters and therefore resolve this issue.

## 5. Conclusions

Based on our previous work of a designed and implemented WHDD, this study performs further static and dynamic experiments to verify the availability and effectiveness of WHDD. In the static experiment, the heart rate and body temperature parameters are corrected by applying the proposed filtering algorithm. In addition, various intensity running experiments are conducted on several runners who wore the WHDD. The experimental results show that the WHDD can successfully identify the high-risk trends of heat stroke when the runners respond to discomfort information, so the device can effectively predict the occurrence of heat stroke and ensure the safety of runners.

## Figures and Tables

**Table 1:** Pathological features and the corresponding physiological risk factors of heat stroke.

Pathological Feature	Physiological Risk Factor	Use of Sensors
Body temperature is always greater than 40.6 °C, heat cannot be dissipated normally from the body	Body temperature	Yes
High-temperature and high-humidity environment	Environmental temperature and humidity	Yes
Lack of sweating or the opposite behavior for heat stroke during sport activity	Skin conductance	Yes
Individuals performing intense exercise	Running	Experimental scenario
Rapid heartbeat or low systolic blood pressure	Heart rate	Yes
Individuals with abnormal BMI, obese individuals, and seniors	Height, weight, and age	Prior investigation

**Table 2:** Comparison of the technical specifications of different low-power wireless communication systems.

Type of Technology	Low-Power WiFi	Bluetooth	LoRa	NB-IoT
Frequency	2.4 GHz 5 GHz	2.4 GHz	868 MHz 915 MHZ	7–900 MHz
Transmission speed	16 Mbps	1 Mbps	12.5 kbps	200 kbps
Transmission power	±17 dBm	±10 dBm	±18 dBm	±23 dBm
Sensitivity	−92 dBm	−89 dBm	−136 dBm	−141 dBm
Transmission range	100 m	30 m	3–15 km	2–20 km
Current consumption (during transmission)	350 mA	22 mA	120 mA	120–300 mA
Standby current	58 μA	1.3 μA	1 μA	5 μA
Topology	Star shape	Star shape	Star shape	Star shape
Node capacity	<30	<30	>10,000	>20,000

**Table 3:** Parameter α for different body parts.

Body Part	*α*
Rectal	0.0699
Head	0.3094
Torso	0.5067
Hand	0.7665
Foot	2.1807

**Table 4:** Physiological differences between users.

User	User 1	User 2	User 3	User 4
**Age**	25	37	23	23
**BMI**	22.9 (normal)	27.3 (mildly obese)	23.2 (upper limit of normal weight)	25 (overweight)
**Exercising habits**	Twice per week	Three times per week	Irregular	Irregular
**Remark**	No exercise before test	Exercise before test	Warm-up before test	Warm-up before test

**Table 5:** Physiological differences between users for outdoor experiment.

User	User 1	User 2	User 3	User 4	User 5
**Age**	37	36	24	24	24
**BMI**	27.3 (mildly obese)	22.8 (normal)	24.0 (upper limit of normal weight)	31 (mildly obese)	23.2 (normal)
**Exercising habits**	Four times per week	Twice per week	Irregular	Irregular	Irregular

**Table 6:** Errors of the heart rates measured in this experiment with respect to data measured using a commercially available sphygmomanometer.

Experimental Runs	Heart Rate (After Filtering)	Average Heart Rate (Commercial Instrument)	Error
1	74	81	−7
2	80	78	2
3	75	80	−5
4	77	75	2
5	73	80	−7
6	91	88	3
7	93	91	2
8	85	78	7
9	84	81	3
10	81	80	1
Average			0.1

**Table 7:** Difference between the body temperature measured in this experiment and that measured using a commercially available infrared temperature gun.

Experimental Runs	Body Temperature (°C) (After Filtering)	Body Temperature (°C) (Commercial Instrument)	Error (°C)
1	36.89	36.3	0.59
2	36.75	36.4	0.35
3	36.33	36.4	−0.07
4	36.85	36.4	0.45
5	36.19	36.5	−0.31
6	36.41	37	−0.59
7	36.59	36.4	0.19
8	36.61	36.5	0.11
9	36.71	36.4	0.31
10	36.81	36.4	0.41
Average			0.14

**Table 8:** Comparison between heart rates measured with the WHDD and with a commercially available heart rate belt during running.

Heart Rate [bpm]	Test Time (min)					
	2	4	6	12	15	Average
WHDD (after filtering)	78	106	140	157	157	131
commercial product	81	125	125	140	155	125
Error	−3	−19	+15	+17	+2	+6

**Table 9:** Comparison between body temperatures measured with WHDD and with a commercially available infrared temperature gun during running.

Body Temperature (°C)	Test Time (min)					
	2	4	6	12	15	Average
WHDD (after filtering)	36.5	36.3	32.4	30.1	30.3	32.3
commercial product	33.8	33.8	32.8	32.5	31.9	32.9
Error	+2.7	+2.5	−0.4	−2.4	−1.6	−0.6

**Table 10:** Comparison between feedback data and system prediction results for user 3.

Testing Time (s)	732		766		804		841	
User	Reported	System	Reported	System	Reported	System	Reported	System
3	Yes	Caution	Yes	Caution	Yes	Caution	Yes	Caution

**Table 11:** Comparison between feedback data and system prediction results for user 4.

Testing Time (s)	816		830		848		851	
User	Reported	System	Reported	System	Reported	System	Reported	System
4	Yes	Dangerous	Yes	Dangerous	Yes	Dangerous	Yes	Dangerous
